# (Bis{2-[3-(2,4,6-trimethyl­benz­yl)imid­azolin-2-yliden-1-yl-κ*C* ^2^]-4-methyl­phenyl}amido-κ*N*)chloridopalladium(II)

**DOI:** 10.1107/S1600536810002382

**Published:** 2010-01-23

**Authors:** Guan-Jun Cheng, Wei Wei, Chuang Zhou, Mei-Ming Luo

**Affiliations:** a Key Laboratory of Green Chemistry and Technology of the Ministry of Education, College of Chemistry, Sichuan University, Chengdu 610064, People’s Republic of China; b School of Bioindustry, Chengdu University, Chengdu 610106, People’s Republic of China

## Abstract

The coordination geometry about the Pd centre in the title compound, [Pd(C_40_H_42_N_5_)Cl], is approximately square-planar. The CNC pincer-type *N*-heterocyclic carbene ligand binds to the Pd atom in a tridentate fashion by the amido N atom and the two carbene atoms and generates two six-membered chelate rings, completing the coordination.

## Related literature

For details of various PNP pincer-type ligands, see: Liang *et al.* (2003 ▶); Fan *et al.* (2004 ▶). For PCP pincer-type ligands, see: Moulton & Shaw (1976 ▶). For general background to pincer-type *N*-heterocyclic carbene ligands and their complexes, see: Moser *et al.* (2007 ▶); Peris *et al.* (2001 ▶). For the catalytic activity of palladium(II) complexes of CNC pincer-type NHC Ligands, see: Loch *et al.* (2002 ▶); Hahn *et al.* (2005 ▶). For the synthesis of the ligand, see: Wei *et al.* (2008 ▶). 
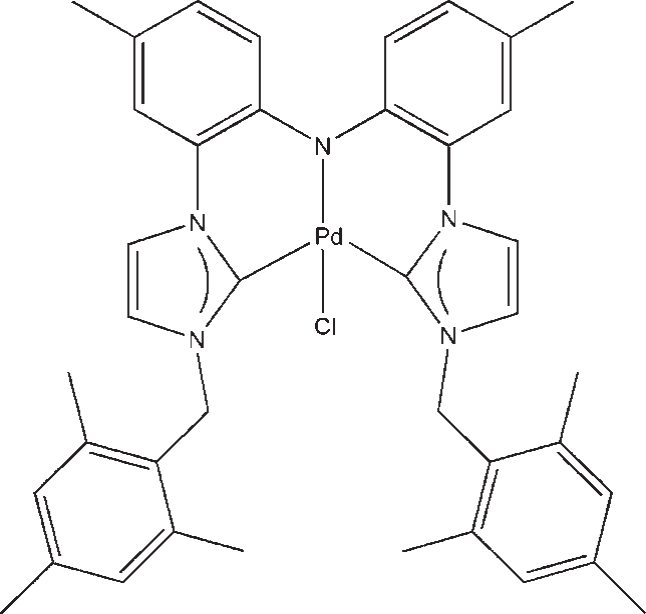


## Experimental

### 

#### Crystal data

[Pd(C_40_H_42_N_5_)Cl]
*M* *_r_* = 734.64
Monoclinic, 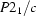

*a* = 14.077 (4) Å
*b* = 28.784 (10) Å
*c* = 10.269 (3) Å
β = 101.87 (3)°
*V* = 4072 (2) Å^3^
*Z* = 4
Mo *K*α radiation
μ = 0.55 mm^−1^
*T* = 295 K
0.45 × 0.40 × 0.12 mm

#### Data collection

Enraf–Nonius CAD-4 diffractometer
Absorption correction: for a sphere (Farrugia, 1999 ▶) *T*_min_ = 0.942, *T*_max_ = 0.984
8356 measured reflections
7268 independent reflections
3639 reflections with *I* > 2σ(*I*)
*R*_int_ = 0.004
3 standard reflections every 300 reflections  intensity decay: 0.4%

#### Refinement

*R*[*F* ^2^ > 2σ(*F* ^2^)] = 0.056
*wR*(*F* ^2^) = 0.148
*S* = 0.97
7268 reflections
436 parameters
H-atom parameters constrained
Δρ_max_ = 0.91 e Å^−3^
Δρ_min_ = −0.68 e Å^−3^

### 

Data collection: *DIFRAC* (Gabe *et al.*, 1993 ▶); cell refinement: *NRCVAX* (Gabe *et al.*, 1989 ▶); data reduction: *NRCVAX*; program(s) used to solve structure: *SHELXS97* (Sheldrick, 2008 ▶); program(s) used to refine structure: *SHELXL97* (Sheldrick, 2008 ▶); molecular graphics: *ORTEP-3 for Windows* (Farrugia, 1997 ▶); software used to prepare material for publication: *SHELXL97* and *PLATON* (Spek, 2009).

## Supplementary Material

Crystal structure: contains datablocks I, global. DOI: 10.1107/S1600536810002382/kj2138sup1.cif

Structure factors: contains datablocks I. DOI: 10.1107/S1600536810002382/kj2138Isup2.hkl

Additional supplementary materials:  crystallographic information; 3D view; checkCIF report

## Figures and Tables

**Fig. 1.**
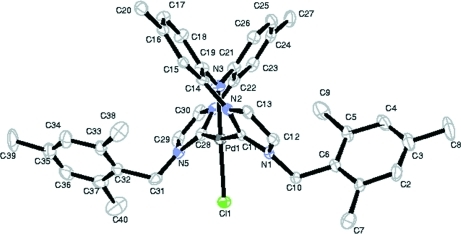
The molecular structure of the title compound with displacement ellipsoids drawn at the 30% probability level.

## supplementary crystallographic information

### Comment

Since the initial report on PCP pincer-type ligands by Moulton and Shaw (1976), a wealth of pincer-type ligands have been reported in recent years (e.g. Peris *et al.*, 2001; Hahn *et al.*, 2005; Moser *et al.*, 2007), owing to their potential for supporting peculiar chemical properties on transition metal centers. Among the phosphine containing pincer-type ligands, the recently emerging PNP ligands with a diarylamido backbone have become attractive due to their unusual reactivity in activation of inert chemical bonds (Liang *et al.*, 2003; Fan *et al.*, 2004; Loch *et al.*, 2002). Therefore, it is surprising to us that no pincer-type bis-NHC ligands based on a diarylamido backbone have been described. Guided by the explorations of the PNP ligand by Liang (Liang *et al.*, 2003), we envisaged that replacement of phosphine arms in the ligand with NHCs would produce new CNC pincer-type ligands that may display useful properties for many challenging catalytic applications, especially for those requiring harsh reaction conditions. We reported the synthesis and catalytic activity of several new pincer-type NHC-Pd complexes (Wei *et al.*, 2008). Though the title compound was synthesized previously by us, the crystal was obtained just recently by growing from dichloromethane and diethyl ether. The crystal structure of the title compound is present here for comparing it with the crystal structure of [bis(2-(3-benzylimidazolin-2-yliden-1-yl)-4-methylphenyl)amido]\ chloropalladium(II) that has been reported earlier (Wei *et al.*, 2008). It is obvious that there are some differences in the coordination geometries at Pd and in the dihedral angles between the two benzene rings of the diarylamido backone and those between the two NHC rings.

The molecular structure of the title compound is depicted in Figure 1. As expected, the monoanionic ligand is coordinated to palladium in a tridentate fashion by the amido nitrogen and the two carbene atoms, forming two six-membered chelate rings. The geometry about Pd is approximately square planar, with the C28—Pd—C11 angle of 170.1 (2)° and the N3–Pd–Cl1 angle of 177.98 (14)°. The two benzene rings of the diarylamido backone form a dihedral angle of 67.50 (16)°. The dihedral angle of the two NHC rings is 80.72 (19)°. There is no H-bond observed in the crystal structure.

### Experimental

A mixture of bis[2-(3-(2,4,6-trimethyl)benzylimidazolium)-4-methylphenyl]amine dibromide (0.100 mmol) and silver(I) oxide (27.6 mg, 0.120 mmol) in 5 ml of solvent (CH_2_Cl_2_/MeCN, V/V=1:1) was stirred at room temperature for 24 h. The reaction mixture was filtered and washed with CH_2_Cl_2_ (10 ml). The combined filtrate was reduced to 5 ml under vacuum. [PdCl_2_(MeCN)_2_] (25.8 mg, 0.100 mmol) in CH_2_Cl_2 _(3 ml) was added to the resulting solution and stirred at room temperature for 2 h. The reaction mixture was filtered and washed with CH_2_Cl_2_ (10 ml). The combined solution was evaporated under reduced pressure to leave a raw product, which was purified by flash chromatography on silica gel (dichloromethane) to give a yellow solid. Yellow single crystals suitable for an X-ray diffraction study were obtained at ambient temperature by slow evaporation of dichloromethane and diethyl ether solution over a period of several days.

### Refinement

All H atom were positioned geometrically with C—H = 0.93, 0.96 and 0.97 Å for aromatic/imidazole, methyl and methylene H and refined using a riding model with displacement parameters of 1.5 *U*_eq_(C) for methyl and *U*_iso_(H) = 1.2 *U*_eq_(C, O) for others. Initial refinements showed the presence of a severely disordered diethylether solvent molecule. Since no satisfactory model could be obtained, the contribution of this disordered density to the final model was taken into account using the SQUEEZE procedure as incorporated in *PLATON* (Spek, 2009). Using this method we found a total number of 39.0, 36.9, 36.8 and 39.1 electrons in each of four symmetry-related cavities with a volume of 209.0, 208.9, 209.0 and 209.0 Å^3^, respectively.

### Crystal data

**Table:**

[Pd(C_40_H_42_N_5_)Cl]	*F*(000) = 1688
*M**_r_* = 734.64	*D*_x_ = 1.198 Mg m^−^^3^
Monoclinic, *P*2_1_/*c*	Mo *K*α radiation, λ = 0.71073 Å
*a* = 14.077 (4) Å	Cell parameters from 23 reflections
*b* = 28.784 (10) Å	θ = 4.5–5.9°
*c* = 10.269 (3) Å	µ = 0.55 mm^−^^1^
β = 101.87 (3)°	*T* = 295 K
*V* = 4072 (2) Å^3^	Block, orange
*Z* = 4	0.45 × 0.40 × 0.12 mm

### Data collection

**Table:**

Enraf–Nonius CAD-4 diffractometer	3639 reflections with *I* > 2σ(*I*)
Radiation source: fine-focus sealed tube	*R*_int_ = 0.004
graphite	θ_max_ = 25.3°, θ_min_ = 1.4°
ω/2–θ scans	*h* = −16→4
Absorption correction: for a sphere (Farrugia, 1999)	*k* = −34→0
*T*_min_ = 0.942, *T*_max_ = 0.984	*l* = −12→12
8356 measured reflections	3 standard reflections every 300 reflections
7268 independent reflections	intensity decay: 0.4%

### Refinement

**Table:**

Refinement on *F*^2^	Primary atom site location: structure-invariant direct methods
Least-squares matrix: full	Secondary atom site location: difference Fourier map
*R*[*F*^2^ > 2σ(*F*^2^)] = 0.056	Hydrogen site location: inferred from neighbouring sites
*wR*(*F*^2^) = 0.148	H-atom parameters constrained
*S* = 0.97	*w* = 1/[σ^2^(*F*_o_^2^) + (0.0702*P*)^2^] where *P* = (*F*_o_^2^ + 2*F*_c_^2^)/3
7268 reflections	(Δ/σ)_max_ = 0.001
436 parameters	Δρ_max_ = 0.91 e Å^−^^3^
0 restraints	Δρ_min_ = −0.68 e Å^−^^3^

### Special details

**Table:**

Geometry. All e.s.d.'s (except the e.s.d. in the dihedral angle between two l.s. planes) are estimated using the full covariance matrix. The cell e.s.d.'s are taken into account individually in the estimation of e.s.d.'s in distances, angles and torsion angles; correlations between e.s.d.'s in cell parameters are only used when they are defined by crystal symmetry. An approximate (isotropic) treatment of cell e.s.d.'s is used for estimating e.s.d.'s involving l.s. planes.
Refinement. Refinement of *F*^2^ against ALL reflections. The weighted *R*-factor *wR* and goodness of fit *S* are based on *F*^2^, conventional *R*-factors *R* are based on *F*, with *F* set to zero for negative *F*^2^. The threshold expression of *F*^2^ > σ(*F*^2^) is used only for calculating *R*-factors(gt) *etc*. and is not relevant to the choice of reflections for refinement. *R*-factors based on *F*^2^ are statistically about twice as large as those based on *F*, and *R*- factors based on ALL data will be even larger.

### Fractional atomic coordinates and isotropic or equivalent isotropic displacement parameters (Å^2^)

**Table:**

	*x*	*y*	*z*	*U*_iso_*/*U*_eq_	
Pd1	0.97388 (4)	0.876856 (14)	0.49293 (4)	0.03970 (15)	
Cl1	0.96935 (11)	0.88082 (5)	0.26123 (13)	0.0465 (4)	
N1	0.8431 (3)	0.96410 (15)	0.4282 (4)	0.0379 (11)	
N2	0.9465 (3)	0.96814 (16)	0.6151 (5)	0.0405 (12)	
N3	0.9755 (4)	0.87133 (16)	0.6870 (4)	0.0482 (13)	
N4	0.9972 (4)	0.77978 (16)	0.5871 (5)	0.0476 (13)	
N5	1.0988 (4)	0.79004 (17)	0.4595 (5)	0.0523 (14)	
C1	0.6102 (5)	0.9848 (2)	0.2470 (6)	0.0542 (17)	
C2	0.5148 (6)	0.9890 (3)	0.2617 (8)	0.078 (2)	
H2	0.4750	1.0109	0.2106	0.094*	
C3	0.4774 (6)	0.9622 (3)	0.3481 (9)	0.079 (2)	
C4	0.5357 (6)	0.9316 (3)	0.4254 (8)	0.082 (2)	
H4	0.5108	0.9139	0.4863	0.098*	
C5	0.6326 (5)	0.9259 (2)	0.4164 (7)	0.0624 (19)	
C6	0.6697 (5)	0.9525 (2)	0.3235 (6)	0.0495 (16)	
C7	0.6444 (6)	1.0151 (3)	0.1469 (7)	0.080 (2)	
H7A	0.6493	0.9970	0.0702	0.120*	
H7B	0.7069	1.0279	0.1857	0.120*	
H7C	0.5989	1.0400	0.1209	0.120*	
C8	0.3729 (6)	0.9674 (4)	0.3621 (10)	0.124 (4)	
H8A	0.3391	0.9386	0.3394	0.186*	
H8B	0.3422	0.9915	0.3034	0.186*	
H8C	0.3712	0.9755	0.4523	0.186*	
C9	0.6969 (7)	0.8911 (3)	0.5102 (9)	0.096 (3)	
H9A	0.7301	0.9071	0.5885	0.145*	
H9B	0.7437	0.8777	0.4652	0.145*	
H9C	0.6570	0.8671	0.5351	0.145*	
C10	0.7742 (4)	0.9455 (2)	0.3112 (5)	0.0429 (14)	
H10A	0.7864	0.9126	0.3023	0.052*	
H10B	0.7848	0.9610	0.2315	0.052*	
C11	0.9146 (4)	0.94019 (18)	0.5071 (5)	0.0352 (13)	
C12	0.8313 (5)	1.00615 (19)	0.4843 (6)	0.0426 (15)	
H12	0.7866	1.0288	0.4478	0.051*	
C13	0.8939 (4)	1.0093 (2)	0.5991 (6)	0.0440 (15)	
H13	0.9012	1.0342	0.6579	0.053*	
C14	1.0110 (4)	0.9543 (2)	0.7358 (5)	0.0412 (14)	
C15	1.0561 (4)	0.98854 (19)	0.8193 (5)	0.0400 (14)	
H15	1.0522	1.0191	0.7891	0.048*	
C16	1.1071 (4)	0.9792 (2)	0.9466 (6)	0.0447 (15)	
C17	1.1103 (5)	0.9332 (2)	0.9870 (6)	0.0534 (17)	
H17	1.1414	0.9258	1.0734	0.064*	
C18	1.0692 (5)	0.8985 (2)	0.9038 (6)	0.0617 (19)	
H18	1.0765	0.8679	0.9333	0.074*	
C19	1.0152 (5)	0.90776 (18)	0.7723 (6)	0.0441 (15)	
C20	1.1546 (5)	1.0174 (2)	1.0386 (6)	0.0592 (18)	
H20A	1.1533	1.0093	1.1289	0.089*	
H20B	1.1200	1.0460	1.0158	0.089*	
H20C	1.2207	1.0211	1.0295	0.089*	
C21	0.9322 (5)	0.83385 (19)	0.7331 (6)	0.0470 (16)	
C22	0.9355 (5)	0.7888 (2)	0.6780 (6)	0.0480 (16)	
C23	0.8818 (5)	0.7527 (2)	0.7169 (7)	0.0576 (18)	
H23	0.8833	0.7238	0.6769	0.069*	
C24	0.8265 (5)	0.7579 (2)	0.8117 (7)	0.0601 (19)	
C25	0.8241 (5)	0.8026 (2)	0.8685 (7)	0.0613 (19)	
H25	0.7878	0.8077	0.9333	0.074*	
C26	0.8754 (5)	0.8384 (2)	0.8281 (6)	0.0570 (18)	
H26	0.8719	0.8675	0.8665	0.068*	
C27	0.7706 (5)	0.7178 (3)	0.8530 (8)	0.084 (3)	
H27A	0.7265	0.7057	0.7765	0.127*	
H27B	0.7346	0.7282	0.9174	0.127*	
H27C	0.8150	0.6938	0.8914	0.127*	
C28	1.0308 (5)	0.81238 (19)	0.5116 (6)	0.0462 (14)	
C29	1.1045 (5)	0.7433 (2)	0.4970 (7)	0.063 (2)	
H29	1.1447	0.7209	0.4718	0.076*	
C30	1.0412 (5)	0.7370 (2)	0.5758 (7)	0.063 (2)	
H30	1.0287	0.7093	0.6157	0.075*	
C31	1.1649 (5)	0.8126 (3)	0.3852 (6)	0.064 (2)	
H31A	1.1484	0.8453	0.3745	0.076*	
H31B	1.1558	0.7989	0.2973	0.076*	
C32	1.2715 (5)	0.8080 (3)	0.4546 (7)	0.0626 (19)	
C33	1.3107 (6)	0.8376 (2)	0.5567 (8)	0.0652 (19)	
C34	1.4079 (6)	0.8326 (3)	0.6198 (9)	0.081 (3)	
H34	1.4348	0.8527	0.6885	0.097*	
C35	1.4637 (7)	0.7986 (4)	0.5818 (11)	0.097 (3)	
C36	1.4247 (7)	0.7711 (4)	0.4771 (10)	0.097 (3)	
H36	1.4645	0.7494	0.4476	0.117*	
C37	1.3280 (7)	0.7739 (3)	0.4119 (8)	0.089 (3)	
C38	1.2505 (7)	0.8746 (3)	0.6079 (9)	0.102 (3)	
H38A	1.2098	0.8899	0.5339	0.153*	
H38B	1.2928	0.8969	0.6599	0.153*	
H38C	1.2108	0.8602	0.6621	0.153*	
C39	1.5697 (6)	0.7926 (4)	0.6577 (12)	0.158 (5)	
H39A	1.6027	0.7714	0.6100	0.237*	
H39B	1.5702	0.7805	0.7449	0.237*	
H39C	1.6020	0.8221	0.6652	0.237*	
C40	1.2915 (8)	0.7407 (4)	0.2986 (9)	0.141 (5)	
H40A	1.3403	0.7177	0.2952	0.212*	
H40B	1.2778	0.7576	0.2163	0.212*	
H40C	1.2335	0.7258	0.3125	0.212*	

### Atomic displacement parameters (Å^2^)

**Table:**

	*U*^11^	*U*^22^	*U*^33^	*U*^12^	*U*^13^	*U*^23^
Pd1	0.0439 (3)	0.0312 (2)	0.0440 (3)	0.0077 (2)	0.00920 (18)	−0.0007 (2)
Cl1	0.0564 (10)	0.0415 (8)	0.0430 (8)	0.0120 (8)	0.0135 (7)	0.0009 (7)
N1	0.031 (3)	0.037 (3)	0.046 (3)	0.000 (2)	0.011 (2)	0.001 (2)
N2	0.040 (3)	0.037 (3)	0.049 (3)	0.002 (2)	0.020 (2)	−0.001 (2)
N3	0.063 (4)	0.037 (3)	0.045 (3)	−0.002 (3)	0.014 (2)	0.001 (2)
N4	0.054 (4)	0.032 (3)	0.050 (3)	0.007 (2)	−0.006 (3)	−0.003 (2)
N5	0.051 (4)	0.040 (3)	0.059 (3)	0.016 (3)	−0.002 (3)	−0.012 (3)
C1	0.036 (4)	0.073 (5)	0.054 (4)	0.018 (4)	0.009 (3)	−0.001 (3)
C2	0.046 (5)	0.102 (7)	0.081 (6)	0.022 (5)	0.001 (4)	−0.015 (5)
C3	0.046 (5)	0.098 (6)	0.096 (6)	−0.004 (5)	0.021 (5)	−0.029 (5)
C4	0.068 (6)	0.096 (6)	0.091 (6)	−0.019 (5)	0.041 (5)	0.000 (5)
C5	0.045 (4)	0.064 (5)	0.083 (5)	−0.008 (4)	0.024 (4)	−0.003 (4)
C6	0.044 (4)	0.053 (4)	0.054 (4)	−0.002 (3)	0.015 (3)	−0.010 (3)
C7	0.069 (6)	0.092 (6)	0.075 (5)	0.031 (5)	0.007 (4)	0.021 (4)
C8	0.040 (5)	0.171 (10)	0.167 (10)	0.000 (6)	0.033 (6)	−0.043 (8)
C9	0.099 (7)	0.068 (5)	0.135 (8)	0.007 (5)	0.052 (6)	0.041 (5)
C10	0.035 (4)	0.053 (4)	0.042 (3)	0.006 (3)	0.010 (3)	0.005 (3)
C11	0.027 (3)	0.035 (3)	0.046 (3)	0.001 (3)	0.012 (3)	0.003 (3)
C12	0.043 (4)	0.035 (3)	0.051 (4)	0.011 (3)	0.015 (3)	0.003 (3)
C13	0.038 (4)	0.042 (4)	0.056 (4)	0.009 (3)	0.022 (3)	0.000 (3)
C14	0.048 (4)	0.046 (3)	0.033 (3)	0.003 (3)	0.016 (3)	0.003 (3)
C15	0.038 (4)	0.040 (3)	0.046 (4)	−0.011 (3)	0.018 (3)	−0.004 (3)
C16	0.045 (4)	0.053 (4)	0.039 (3)	−0.005 (3)	0.015 (3)	−0.011 (3)
C17	0.061 (5)	0.054 (4)	0.044 (4)	0.002 (3)	0.008 (3)	−0.002 (3)
C18	0.072 (5)	0.052 (4)	0.055 (4)	−0.004 (4)	−0.002 (4)	0.000 (3)
C19	0.064 (5)	0.027 (3)	0.043 (3)	0.015 (3)	0.016 (3)	0.004 (3)
C20	0.051 (5)	0.067 (4)	0.062 (4)	−0.015 (4)	0.017 (3)	−0.018 (3)
C21	0.058 (4)	0.034 (3)	0.046 (4)	0.009 (3)	0.004 (3)	0.010 (3)
C22	0.052 (4)	0.034 (3)	0.051 (4)	0.003 (3)	−0.005 (3)	0.005 (3)
C23	0.058 (5)	0.032 (3)	0.073 (5)	−0.003 (3)	−0.008 (4)	0.007 (3)
C24	0.041 (4)	0.055 (4)	0.076 (5)	−0.009 (3)	−0.007 (4)	0.022 (4)
C25	0.050 (5)	0.061 (5)	0.073 (5)	0.009 (4)	0.014 (4)	0.019 (4)
C26	0.060 (5)	0.048 (4)	0.061 (4)	0.000 (3)	0.006 (4)	0.012 (3)
C27	0.050 (5)	0.091 (6)	0.106 (6)	−0.019 (4)	0.002 (4)	0.029 (5)
C28	0.042 (4)	0.039 (3)	0.055 (4)	0.010 (3)	0.002 (3)	−0.008 (3)
C29	0.060 (5)	0.040 (4)	0.080 (5)	0.014 (4)	−0.011 (4)	−0.016 (4)
C30	0.062 (5)	0.030 (3)	0.086 (5)	0.004 (3)	−0.010 (4)	−0.010 (3)
C31	0.066 (5)	0.075 (5)	0.047 (4)	0.019 (4)	0.007 (4)	−0.010 (3)
C32	0.048 (5)	0.083 (5)	0.058 (4)	0.021 (4)	0.014 (3)	0.014 (4)
C33	0.056 (5)	0.058 (5)	0.081 (5)	0.000 (4)	0.013 (4)	0.015 (4)
C34	0.058 (6)	0.059 (5)	0.121 (7)	−0.017 (4)	0.005 (5)	0.036 (5)
C35	0.064 (7)	0.113 (8)	0.114 (8)	0.000 (6)	0.016 (6)	0.063 (7)
C36	0.066 (6)	0.138 (9)	0.097 (7)	0.049 (6)	0.038 (5)	0.034 (6)
C37	0.084 (7)	0.122 (7)	0.065 (5)	0.046 (6)	0.025 (5)	0.006 (5)
C38	0.114 (8)	0.056 (5)	0.121 (7)	−0.002 (5)	−0.010 (6)	−0.016 (5)
C39	0.038 (5)	0.205 (12)	0.218 (12)	0.010 (7)	−0.005 (7)	0.100 (10)
C40	0.160 (11)	0.159 (10)	0.098 (7)	0.090 (8)	0.011 (7)	−0.035 (7)

### Geometric parameters (Å, °)

**Table:**

Pd1—N3	1.995 (5)	C16—C17	1.386 (8)
Pd1—C28	2.015 (6)	C16—C20	1.512 (8)
Pd1—C11	2.022 (5)	C17—C18	1.364 (8)
Pd1—Cl1	2.3697 (16)	C17—H17	0.9300
N1—C11	1.345 (7)	C18—C19	1.431 (8)
N1—C12	1.365 (6)	C18—H18	0.9300
N1—C10	1.480 (7)	C20—H20A	0.9600
N2—C11	1.369 (7)	C20—H20B	0.9600
N2—C13	1.388 (7)	C20—H20C	0.9600
N2—C14	1.434 (7)	C21—C26	1.388 (9)
N3—C21	1.370 (7)	C21—C22	1.419 (8)
N3—C19	1.407 (7)	C22—C23	1.391 (8)
N4—C28	1.362 (8)	C23—C24	1.375 (9)
N4—C30	1.394 (7)	C23—H23	0.9300
N4—C22	1.423 (8)	C24—C25	1.416 (9)
N5—C28	1.352 (8)	C24—C27	1.507 (9)
N5—C29	1.397 (8)	C25—C26	1.371 (9)
N5—C31	1.470 (9)	C25—H25	0.9300
C1—C6	1.383 (8)	C26—H26	0.9300
C1—C2	1.386 (9)	C27—H27A	0.9600
C1—C7	1.501 (9)	C27—H27B	0.9600
C2—C3	1.362 (11)	C27—H27C	0.9600
C2—H2	0.9300	C29—C30	1.333 (10)
C3—C4	1.346 (11)	C29—H29	0.9300
C3—C8	1.514 (10)	C30—H30	0.9300
C4—C5	1.397 (10)	C31—C32	1.530 (9)
C4—H4	0.9300	C31—H31A	0.9700
C5—C6	1.405 (9)	C31—H31B	0.9700
C5—C9	1.546 (10)	C32—C33	1.376 (9)
C6—C10	1.516 (8)	C32—C37	1.388 (10)
C7—H7A	0.9600	C33—C34	1.396 (10)
C7—H7B	0.9600	C33—C38	1.520 (11)
C7—H7C	0.9600	C34—C35	1.362 (12)
C8—H8A	0.9600	C34—H34	0.9300
C8—H8B	0.9600	C35—C36	1.357 (13)
C8—H8C	0.9600	C35—C39	1.544 (12)
C9—H9A	0.9600	C36—C37	1.391 (12)
C9—H9B	0.9600	C36—H36	0.9300
C9—H9C	0.9600	C37—C40	1.511 (12)
C10—H10A	0.9700	C38—H38A	0.9600
C10—H10B	0.9700	C38—H38B	0.9600
C12—C13	1.322 (8)	C38—H38C	0.9600
C12—H12	0.9300	C39—H39A	0.9600
C13—H13	0.9300	C39—H39B	0.9600
C14—C15	1.374 (7)	C39—H39C	0.9600
C14—C19	1.389 (7)	C40—H40A	0.9600
C15—C16	1.382 (8)	C40—H40B	0.9600
C15—H15	0.9300	C40—H40C	0.9600
			
N3—Pd1—C28	84.8 (2)	C19—C18—H18	119.1
N3—Pd1—C11	85.4 (2)	C14—C19—N3	124.1 (5)
C28—Pd1—C11	170.1 (2)	C14—C19—C18	114.8 (5)
N3—Pd1—Cl1	177.98 (14)	N3—C19—C18	120.9 (5)
C28—Pd1—Cl1	93.89 (19)	C16—C20—H20A	109.5
C11—Pd1—Cl1	95.88 (15)	C16—C20—H20B	109.5
C11—N1—C12	109.9 (5)	H20A—C20—H20B	109.5
C11—N1—C10	126.1 (5)	C16—C20—H20C	109.5
C12—N1—C10	123.1 (5)	H20A—C20—H20C	109.5
C11—N2—C13	109.1 (5)	H20B—C20—H20C	109.5
C11—N2—C14	125.5 (5)	N3—C21—C26	121.9 (5)
C13—N2—C14	124.5 (5)	N3—C21—C22	121.8 (6)
C21—N3—C19	121.3 (5)	C26—C21—C22	116.0 (6)
C21—N3—Pd1	119.6 (4)	C23—C22—C21	120.2 (6)
C19—N3—Pd1	119.0 (4)	C23—C22—N4	119.5 (6)
C28—N4—C30	110.4 (6)	C21—C22—N4	120.2 (6)
C28—N4—C22	125.3 (5)	C24—C23—C22	122.9 (6)
C30—N4—C22	123.8 (6)	C24—C23—H23	118.5
C28—N5—C29	110.7 (6)	C22—C23—H23	118.5
C28—N5—C31	124.7 (5)	C23—C24—C25	117.0 (6)
C29—N5—C31	124.3 (6)	C23—C24—C27	121.4 (7)
C6—C1—C2	118.9 (7)	C25—C24—C27	121.6 (7)
C6—C1—C7	122.5 (6)	C26—C25—C24	120.0 (7)
C2—C1—C7	118.6 (7)	C26—C25—H25	120.0
C3—C2—C1	122.3 (7)	C24—C25—H25	120.0
C3—C2—H2	118.8	C25—C26—C21	123.8 (6)
C1—C2—H2	118.8	C25—C26—H26	118.1
C4—C3—C2	119.0 (7)	C21—C26—H26	118.1
C4—C3—C8	119.7 (9)	C24—C27—H27A	109.5
C2—C3—C8	121.3 (9)	C24—C27—H27B	109.5
C3—C4—C5	121.6 (7)	H27A—C27—H27B	109.5
C3—C4—H4	119.2	C24—C27—H27C	109.5
C5—C4—H4	119.2	H27A—C27—H27C	109.5
C4—C5—C6	119.0 (7)	H27B—C27—H27C	109.5
C4—C5—C9	119.3 (7)	N5—C28—N4	104.7 (5)
C6—C5—C9	121.6 (6)	N5—C28—Pd1	134.5 (5)
C1—C6—C5	119.1 (6)	N4—C28—Pd1	120.7 (5)
C1—C6—C10	121.8 (6)	C30—C29—N5	107.0 (6)
C5—C6—C10	119.1 (6)	C30—C29—H29	126.5
C1—C7—H7A	109.5	N5—C29—H29	126.5
C1—C7—H7B	109.5	C29—C30—N4	107.0 (6)
H7A—C7—H7B	109.5	C29—C30—H30	126.5
C1—C7—H7C	109.5	N4—C30—H30	126.5
H7A—C7—H7C	109.5	N5—C31—C32	112.6 (5)
H7B—C7—H7C	109.5	N5—C31—H31A	109.1
C3—C8—H8A	109.5	C32—C31—H31A	109.1
C3—C8—H8B	109.5	N5—C31—H31B	109.1
H8A—C8—H8B	109.5	C32—C31—H31B	109.1
C3—C8—H8C	109.5	H31A—C31—H31B	107.8
H8A—C8—H8C	109.5	C33—C32—C37	120.8 (7)
H8B—C8—H8C	109.5	C33—C32—C31	120.1 (6)
C5—C9—H9A	109.5	C37—C32—C31	119.1 (7)
C5—C9—H9B	109.5	C32—C33—C34	119.4 (8)
H9A—C9—H9B	109.5	C32—C33—C38	122.2 (7)
C5—C9—H9C	109.5	C34—C33—C38	118.3 (8)
H9A—C9—H9C	109.5	C35—C34—C33	120.6 (9)
H9B—C9—H9C	109.5	C35—C34—H34	119.7
N1—C10—C6	111.7 (5)	C33—C34—H34	119.7
N1—C10—H10A	109.3	C36—C35—C34	118.9 (9)
C6—C10—H10A	109.3	C36—C35—C39	121.7 (11)
N1—C10—H10B	109.3	C34—C35—C39	119.4 (11)
C6—C10—H10B	109.3	C35—C36—C37	123.0 (9)
H10A—C10—H10B	107.9	C35—C36—H36	118.5
N1—C11—N2	105.5 (5)	C37—C36—H36	118.5
N1—C11—Pd1	133.4 (4)	C32—C37—C36	117.2 (9)
N2—C11—Pd1	121.1 (4)	C32—C37—C40	124.3 (8)
C13—C12—N1	108.6 (5)	C36—C37—C40	118.5 (8)
C13—C12—H12	125.7	C33—C38—H38A	109.5
N1—C12—H12	125.7	C33—C38—H38B	109.5
C12—C13—N2	106.8 (5)	H38A—C38—H38B	109.5
C12—C13—H13	126.6	C33—C38—H38C	109.5
N2—C13—H13	126.6	H38A—C38—H38C	109.5
C15—C14—C19	122.3 (5)	H38B—C38—H38C	109.5
C15—C14—N2	118.0 (5)	C35—C39—H39A	109.5
C19—C14—N2	119.1 (5)	C35—C39—H39B	109.5
C14—C15—C16	122.3 (5)	H39A—C39—H39B	109.5
C14—C15—H15	118.8	C35—C39—H39C	109.5
C16—C15—H15	118.8	H39A—C39—H39C	109.5
C15—C16—C17	116.5 (5)	H39B—C39—H39C	109.5
C15—C16—C20	121.8 (6)	C37—C40—H40A	109.5
C17—C16—C20	121.6 (6)	C37—C40—H40B	109.5
C18—C17—C16	122.0 (6)	H40A—C40—H40B	109.5
C18—C17—H17	119.0	C37—C40—H40C	109.5
C16—C17—H17	119.0	H40A—C40—H40C	109.5
C17—C18—C19	121.9 (6)	H40B—C40—H40C	109.5
C17—C18—H18	119.1		
